# Virological and serological surveillance for type A influenza in the black-legged kittiwake *(Rissa tridactyla)*

**DOI:** 10.1186/1743-422X-8-21

**Published:** 2011-01-17

**Authors:** Ragnhild Toennessen, Anna Germundsson, Christine M Jonassen, Irene Haugen, Kristin Berg, Robert T Barrett, Espen Rimstad

**Affiliations:** 1 Department of Food Safety & Infection Biology, Norwegian School of Veterinary Science, P. O. Box 8146 Dep, N-0033 Oslo, Norway; 2 Norwegian Veterinary Institute, P. O. Box 750 Dep, N-0106 Oslo, Norway; 3 Center for Laboratory Medicine, Akershus University Hospital, N-1478 Lørenskog, Norway; 4 Department of Natural Sciences, Tromsø University Museum, N-9037 Tromsø, Norway

## Abstract

**Background:**

The epidemiology of avian influenza viruses (AIVs) in gulls is only partially known. The role of the world's most numerous gull species, the black-legged kittiwake (*Rissa tridactyla*), as a potential AIV reservoir species has been unclear. The prevalence of AIV and humoral response against AIV were therefore studied in a colony of apparently healthy black-legged kittiwakes breeding in a nesting cliff in the South West Barents Region of Norway (70°22' N, 31°10' E), in 2008 and 2009.

**Results:**

AIVs were detected from the oropharynx and cloaca in low amounts, with prevalences of 15% and 5%, in 2008 and 2009, respectively. Direct, partial sequencing of the hemagglutinin (HA) gene revealed that the H4 subtype was present. In 2009, antibodies to influenza A virus were detected in sera from 57 of 80 adult birds. In contrast, none of the three-week-old chicks (n = 18) tested seropositive. Hemagglutination inhibition (HI) assays demonstrated that the adult kittiwakes primarily had antibodies specific to the gull-associated H13 and H16 subtypes, with antibodies to H16 being most common.

**Conclusions:**

These results support that the highly pelagic black-legged kittiwake is a reservoir of AIV. The serological findings suggest that H16 might be the main AIV subtype in the black-legged kittiwake. Further studies are needed to understand the ecology of AIV in the black-legged kittiwake and in gulls in general.

## Background

Wild birds in the orders Anseriformes (ducks, geese and swans) and Charadriiformes (gulls, terns and shorebirds) collectively are the natural reservoir for all known subtypes of avian influenza viruses (AIVs) [1,2]. The outbreaks of highly pathogenic (HP) AIV subtype H5N1 in Southeast Asia emphasized the importance of studying the dynamics of AIV infections in relation to the ecology of the natural hosts [3]. The global surveillance programs for the Eurasian HPAIV H5N1 in wild birds have contributed to increased knowledge about low pathogenic (LP) AIVs, particularly in ducks [4]. However, the epidemiology of AIV infections in most gull species is still only partially known [5,6].

HPAI in wild birds was first detected in common terns (*Sterna hirundo*) in South Africa, 1961 [7]. Since then, AIVs, including Eurasian HPAI H5N1 [8-10], have been detected in several gull (Laridae) species [3]. The AIV prevalence in gulls has generally been found to be low [11].

Influenza virus subtypes H13 and H16 seem to be gull-associated [2,12] and have been suggested to represent a unique gene pool of AIVs that differs from that of waterfowl [13]. Except for a single isolation of AIV subtype H13N9 from a kelp gull (*Larus dominicanus*) in South America [14], H13 and H16 influenza viruses have so far only been detected in the northern hemisphere [3,15-19] with H13 being the most common [20]. Influenza A virus of the H13 subtype was first reported from ring-billed (*Larus delawarensis*), Franklin's (*Leucophaeus pipixcan*), great black-backed (*Larus marinus*) and herring gulls (*Larus argentatus*) in the United States in 1982 [12] and has also been isolated from pilot whale (*Globicephala melaena*) [21]. Recently, homologue subtype H13N9 AIVs were detected from two glaucous gulls (*Larus hyperboreus*) and a lesser snow goose (*Chen caerulescens*) in Alaska [16], indicating that sharing of habitat might be important for virus transmission within and between bird species. The closely related H16 subtype was reported first in 2005 from black-headed gulls (*Chroicocephalus ridibundus*) in Sweden [2] and has until now primarily been detected in gulls and shorebirds [19].

High antibody prevalences to influenza A virus that have been found in several gull species from North America [22], and substantial proportion of gene reassortment between Eurasian and North American AIVs found in Laridae in Alaska where migratory flyways overlap [16], point to the importance of studying the role of gulls in the epidemiology of AI.

With 6-7 million breeding pairs, the black-legged kittiwake (*Rissa tridactyla*), a cliff-nesting gull species of the Laridae family, is the most numerous gull species in the world [23]. In contrast to the much studied and more easily caught ducks that largely inhabit wetlands in close proximity to humans, the highly pelagic kittiwake has a circumpolar distribution and breeds in the Boreal and Arctic zones in the northern hemisphere [23].

In North America, a large-scale retrospective sequencing study of influenza isolates from wild birds showed that a black-legged kittiwake, sampled in Alaska in 1975, was infected with H16N3 [19,24] indicating that the H16 subtype had been present a long time before its first description in 2005 [2].

Several screening studies of AIV in wild birds where small numbers of kittiwakes have been included, have not resulted in any detection of influenza viruses in this species [3,15,25,26]. To our knowledge, there are no reports on the humoral response against AIV in kittiwakes, neither has AIV been detected in black-legged kittiwakes in Europe. With only one single previous isolation of AIV from a black-legged kittiwake, the role of the species as a reservoir for AIV is still unclear. The aim of this study was to examine the prevalence of AIV and the humoral response against AIV, to study the role of the kittiwake as an AIV reservoir species.

## Methods

### Study design

Two cross-sectional studies were conducted, in 2008 and 2009 respectively, at the island Hornøya (70°22' N, 31°10' E) in the South West Barents Sea, in the northern part of Norway. More than 10 000 pairs of black-legged kittiwakes breed in this densely populated seabird colony from April to September each year in close proximity to common murres (*Uria aalge*), herring gulls (*Larus argentatus*), razorbills (*Alca torda*) Atlantic puffins (*Fratercula arctica*), European shags (*Phalacrocorax aristotelis*), black guillemots (*Cepphus grylle*) and great black-backed gulls (*Larus marinus*) (R.T. Barrett, personal communication).

In the beginning of July 2008, swab samples were collected during ringing from 100 apparently healthy, adult black-legged kittiwakes nesting in a cliff colony. The birds were caught on their nests with a noose pole, marked to prevent recapture, and released after sampling. Oropharyngeal and cloacal swabs, using sterile cotton swabs (SelefaTrade^®^, Spånga, Sweden), were sampled from each bird, and pooled in 1.5 ml virus transport medium (Eagle's Minimum Essential Medium supplemented with 2% fetal bovine serum and 100 μg/ml gentamicin). The samples were stored cool until the analysis started within three days.

In the end of June 2009, oropharyngeal and cloacal samples were collected from 80 adults and 20 approximately three-week-old chicks, using nylon flocked swabs (Copan Innovation, Brescia, Italy). The chicks were caught by hand in their nests, aged based on plumage development [27] and returned to their nest after sampling. All birds were weighed and ring numbers were registered. The oropharyngeal and cloacal swabs from each bird were put in separate tubes containing 1.5 ml Universal Transport Medium (Copan Innovation, Brescia, Italy) each. The analysis of the swab samples started within maximum five days post sampling. Blood samples were collected from both chicks and adult birds in 2009.

The software EpiCalc 2000 version 1.02 [28] was used to estimate the number of birds to sample. The birds were caught with permission from the Norwegian Directorate for Nature Management and sampling methods were approved by the Norwegian Animal Research Authority (NARA). A permission to work in the nature reserve at Hornøya was granted from the county governor of Finnmark.

### rRT- PCR

RNA was extracted from 200 μl medium from each swab sample using the automatic extraction system NucliSens^® ^easyMag™ (bioMérieux, Boxtel, The Netherlands) according to the manufacturer's protocol. The rest of the swab samples were stored at -20°C in 2008 and -80°C in 2009. An initial screening for AIV using a real-time reverse transcriptase polymerase chain reaction (rRT-PCR) with primers and probe targeting the conserved part of the matrix (M) gene was performed [29]. Samples with cycle threshold (Ct) values <40 were considered positive [30]. AIV positive samples were tested with a H5-specific rRT-PCR [31]. In each PCR run, RNA from a LPAI H5N3 isolate (SVA1174/05), obtained from the National Veterinary Institute of Sweden, was used as positive control, while RNA from the negative RNA extraction control (NucliSens^® ^lysis buffer) was used as negative control.

### Sequencing and genetic analysis

The AIV positive samples were also tested in a SYBR green real-time PCR targeting a larger part of the M gene [32]. In order to confirm AIV, the M amplicons were purified using the PCR-M™ Clean Up System (Viogene, Sunnyvale, CA, USA) and sequenced in both directions using a commercial sequencing service (GATC Biotech, Constance, Germany).

All samples positive for influenza A virus, but negative for subtype H5, were further HA subtyped by performing RT-PCRs for the HA2 [33]. The HA2 amplicons were sequenced using ABI PRISM BigDye terminator cycle sequencing ready reaction kit version 3.1 (Applied Biosystem, Foster City, CA, USA) according to the manufacturer's instructions and analysed on an ABI PRISM 3100-*Avant *Genetic Analyzer (Applied Biosystems, Foster City, CA, USA). The Vector NTI Advance™ 11 software (Invitrogen, Carlsbad, CA, USA) was used for sequence analysis. Sequence similarity searches were performed using BLAST [34].

### Virus isolation and hemagglutination assay

Transport media from swab samples were thawed, vortexed, briefly centrifuged and 12.5-25 μl Gentamicin (50 mg/ml, GIBCO^®^) added to the supernatant. After approximately 1 h incubation at room temperature (RT), 100-200 μl medium from each sample was inoculated into the allantoic cavity of 9-11 days old specific antibody negative embryonated chicken eggs. Presence of hemagglutinating agents was determined by the hemagglutination assay using 1% chicken erythrocytes [35].

### Blood samples

Blood samples of approximately 1-2 ml were taken from the brachial vein of the adult birds (n = 80) using 2 ml BD Vacutainers^® ^with no additive (Z), while 0.1-0.2 ml blood samples were collected from the chicks using 1 ml syringes (Terumo^®^) with heparin-treated 25G needles. Due to poor serum outcome from two of the blood samples, serum samples were only obtained from 18 of the 20 chicks. The blood samples were allowed to clot at RT for 24 h, before sera were collected and stored at 4°C for up to four days, until frozen at -20°C when arriving at the laboratory.

### ELISA and hemagglutination inhibition (HI) assay

The serum samples were diluted 1:10 and screened for antibodies specific to the influenza A virus nucleoprotein (NP) using a Competitive Enzyme-Linked Immunosorbent Assay (cELISA) (ID-screen^® ^Influenza A Antibody Competition ELISA kit, IDVet, Montpellier, France) following the manufacturer's instructions.

Serum samples positive in the cELISA with a residual volume of ≥ 70 μl still available, were further screened in an HI assay as previously described [36]. To remove possible non-specific hemagglutinins, samples were diluted 1:10 with PBS de Boer and incubated with chicken erythrocytes at a final concentration of 1% (v/v) for 30 minutes at RT with inversion of the tubes every 5 minutes. After centrifugation at 2500 rpm/5 minutes, the supernatants were screened in the HI assay using H4, H5, H13 and H16 as antigens. A HI titre of ≥ 1:20 was considered as positive. The antigens and antibodies used in the HI assay are listed in Table 1.

**Table 1:** Antigens and antibodies used in the HI assay

Antigens	Antibodies
A/Mallard/Norway/10_1334/2006 (H4N6)	A/Duck/Czeck/56 (H4N6)
A/Chicken/Scotland/1959 (H5N1)	A/Turkey/Turkey/05 (H5N1)
A/Ostrich/Denmark/72420/96 (H5N2)	A/Ostrich/Denmark/72420/96 (H5N2)
A/Tern/South Africa/61 (H5N3)	A/Tern/South Africa/61 (H5N3)
A/Common gull/Norway/10_1313/2009( H13N2)	N/A
A/Herring gull/Norway/10_2336/2006 (H13N6)	A/Gull/Maryland/704/77 (H13N6)
A/Common gull/Norway/10_1617/2006 (H16N3)	A/Gull/Denmark/68110/02 (H16N3)

The virus reference antigens and antibodies used in the HI assay were obtained from the Veterinary Laboratories Agency (Weybridge, England) and the Norwegian Veterinary Institute, (Oslo, Norway). N/A= Not available.

## Results

### Virology

Influenza A virus RNA was detected in 15% (n = 15) of the adult birds in 2008 and in 5% (n = 4) of the adult birds and 5% (n = 1) of the chicks in 2009 using rRT-PCR targeting the M gene [29]. None of the samples were positive for the H5 subtype. In 2009, oropharyngeal and cloacal swabs were analysed separately. From the adult birds, viral RNA was detected in two oropharyngeal swab samples (Ct values 35.6 and 37.4) and in two cloacal samples (Ct values 36.5 and 38.9). In addition, one swab sample from the cloaca of a three-week-old chick tested positive (Ct value 37.9). None of the birds were positive for AIV in both the cloacal and oropharyngeal swab. The average Ct value for the positive samples was 37.1 and ranged from 32.2 to 39.8 in 2008. Three of the adult kittiwakes that were AIV positive in 2008, were recaptured and found to be AIV negative in 2009.

None of the swab samples positive for influenza A virus by rRT-PCR yielded any virus isolates after inoculation of transport media in embryonated eggs.

In 2008, HA subtyping by sequencing resulted in detection of H4 in one sample and partial M sequences were obtained from four samples found positive in the SYBR green PCR. The partial H4 sequence (188 bp) showed highest similarity to Eurasian avian-like H4 viruses. Only two of the four obtained M sequences (both 200 bp) had optimal sequence quality and were therefore submitted to GenBank [GenBank: FR687037-38]. Sequence comparison showed that the two M sequences were most similar to an AIV subtype H13N6 isolated from a Mongolian gull in Mongolia, 2007 [GenBank: GQ907319] (100% nucleotide (nt) identity) and an AIV subtype H16N3 isolated from a black-headed gull in Sweden, 1999 [GenBank: AY684908] (98% nt identity), respectively.

### Serology

**Figure 1:**
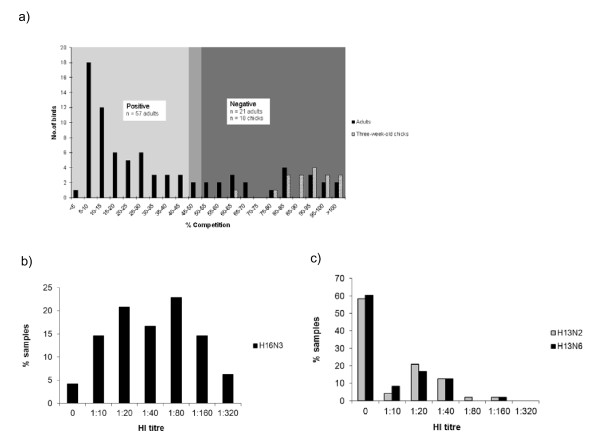
**cELISA and HI assay**. Results from the cELISA for detection of antibodies to influenza A virus NP in black-legged kittiwake sera collected in 2009. Sera from adults (n = 80) and three-week old chicks (n = 18) were tested. A result was defined as positive when the competition was ≤ 45%, doubtful at 45-50% and negative at ≥ 50% competition (a). The distribution of HI titres against AIV subtypes H16 (b) and H13 (c) in sera from black-legged kittiwakes (n = 48).

Blood samples were collected in 2009. In total, 57 of 80 (71.3%) adult birds were AIV seropositive. In contrast, all the 18 chicks were seronegative (Figure 1a). Of the recaptured birds, only one out of three birds that were rRT-PCR AIV positive in 2008 tested positive in the cELISA.

Of the 57 samples that tested positive in the cELISA test, 48 had a sufficient residual volume to be tested in the HI assay. None of the birds had HI antibodies specific to H4 and H5, while HI antibodies specific to H13 and H16 were detected in 37.5% (18/48) and 81.3% (39/48) of the samples, respectively. In 43.8% (21/48) of the samples, only HI antibodies to H16 were found, while 37.5% (18/48) contained antibodies to both H13 and H16 AIVs. None of the birds (0/48) had antibodies to H13 AIV, exclusively. The distributions of the HI titres to H16 and H13 AIVs are presented in Figure 1b) and 1c). The geometric mean antibody titres for H13N2, H13N6 and H16N3 were 27.3, 30.3 and 61.3, respectively. Nine samples that tested positive in the cELISA tested negative (defined as HI titre <1:20) for the antigens used in the HI assay. Seven of these had antibody titres of 1:10 to H16 AIV, while the remaining two samples were negative.

## Discussion

Low AIV loads were detected in black-legged kittiwakes at a nesting cliff, Hornøya in the Barents Sea, Norway, at the beginning of the chick-rearing period. Therefore, the AIV prevalence in the nesting areas could not be used as an aid for drawing conclusions regarding the epidemiology and the course of infection. However, serology indicated that a large proportion of the adult kittiwakes had been infected with AIV, subtype H16. The AIV prevalence in the kittiwakes varied between the two years of sampling and was higher than the prevalence reported from gulls in general [11], but lower than that reported from ring-billed gull colonies in Canada [6]. In Norway, the total AIV prevalence in swabs from gulls (i.e. mew gull (*Larus canus*), herring gull, black-headed gull, lesser black-backed gull (*Larus fuscus*), great black-backed gull and black-legged kittiwake) collected for AIV surveillance during fall 2006 and 2007, was 6.1% [15].

Low virus loads as indicated by high Ct values, as well as suboptimal sample storage conditions could be of importance for the lack of success of virus isolation. In addition the discordant virus isolation and PCR results could be caused by the freeze thaw cycles between the tests. A substantial fraction of samples from wild birds commonly give high Ct values for AIV and it is well known that it is difficult to isolate virus from such samples and also from samples stored at -20°C compared to samples stored at -80°C [30]. Limited viral shedding could also contribute to the low viral amounts observed in the samples.

In ducks, AIV has a fecal-oral transmission route [37]. Breeding colonies for gulls represent a feces-rich environment and have been suggested to be a likely place for virus transmission in ring-billed gulls [6]. Our results from two following years did not indicate extensive spread of AIV in the colony at the time of sampling. Over multiple years in The Netherlands and Sweden, Influenza A virus was undetectable in many different gull colonies during breeding season, while an AIV prevalence of 60% was detected in juvenile black-headed gulls in August in Sweden, i.e. during fall migration. Munster et al. therefore suggested that the prevalence of AIV peaked shortly after the gulls had left the breeding grounds [3]. The pelagic kittiwake spends the first three to five years of life offshore where it can travel long distances, before returning, often to the same breeding colony where it was hatched [38]. In this period, kittiwakes are difficult to survey and AIV infections would go unnoticed.

Recoveries of kittiwakes ringed in northern Norway show that when the birds leave the colony after the breeding season they disperse over huge areas of the North Atlantic, from Newfoundland in the west to the Baltic and North Seas in the east. Some may even enter the Mediterranean Sea [39,40]. This dispersal pattern is common for all European kittiwakes such that birds originating from many geographical areas are likely to be present simultaneously in the same areas and even mix with birds from North America and Greenland when being in the New World. Young kittiwakes tend to remain far from the colonies in their first winter and second summer whereas older birds were recovered closer to the breeding areas in northern Norway and the southern Barents Sea. Although widely dispersed, kittiwakes, like many other seabirds, may form dense flocks when feeding on isolated food patches.

In our study, one kittiwake was found to be infected with the H4 subtype, but due to the low viral load in the sample, only a partial HA sequence was obtained. In Europe, including Norway, AIV H4 is one of the most common subtypes isolated from wild birds [3,15]. The H4 AIVs have been frequently detected from ducks, but rarely from other birds in northern Europe [3]. In Norway, the H4 subtype has occasionally been detected from black-headed gulls and great black-backed gulls [15]. The results from the HI assay did not indicate that antibodies to H4 were common in the kittiwake population under study. Although other AIV subtypes than H13 and H16 have been detected in gulls [15,41-44], it has been proposed that viruses that are genetically indistinguishable from viruses of other avian hosts are not endemic in gulls [20]. Further investigations are necessary to determine if the kittiwake is a natural host for the H4 AIVs or if it could have been coincidentally transmitted through mixing with other seabirds in the densely populated colony.

The results of the cELISA showed a prevalence of 71.3% of anti-AIV antibodies, which is similar to the moderate (40-45%) to high AIV antibody prevalence (>80%) found by Brown et al. in sera from gull species in North America [22]. In Iran, antibodies to AIV were found in 28.6% (4/14) of gulls tested between October and March 2003-2007, all positives were black-headed gulls (4/10; 40%) [42].

Limited data exist for the use of sera from wild birds in the cELISA in general, and in gulls specifically [45]. It is difficult to validate the cELISA on wild birds due to unknown infection status, but a comparable ELISA, also based on monoclonal antibodies produced in mice, has recently been assessed as a sensitive and reliable tool in studies of AIV epidemiology [22]. The curve in Figure 1a) indicates a peak in prevalence of positive sera at about 5-10% competition, followed by a decline in prevalence giving the curve a typical tail appearance. The cut off at 50% is well within this tail, indicating that the calculated seroprevalence could be an underestimation.

Of the recaptured birds, only one out of three birds that were rRT-PCR AIV positive in 2008 tested positive in the cELISA in 2009. The duration of the humoral immune response to AIV infections in kittiwakes is unknown. Infection with AIV that produced a weak and short lasting humoral response or no detectable humoral immunoglobulin Y (IgY) response at all might be an explanation for the two cELISA negative birds. If this is the case, the real AIV infection prevalence in kittiwakes could be even higher than the results found by using the cELISA.

All three-week-old chicks examined, were negative for antibodies to influenza A virus. Transfer of AIV specific maternal antibodies has been documented in yellow-legged gulls (*Larus michahellis*) [46]. In kittiwakes maternally derived IgY is catabolised within two weeks post hatching [47] and the chicks should be susceptible to infection thereafter. Small amounts of viral RNA were detected in the cloacal swab from one chick in 2009 which tested negative in the cELISA. As viral RNA was only detected in 5% of the adult kittiwakes in 2009, one would not expect to find a large number of infected chicks with the given sample size. A recent infection without time to develop a detectable humoral response could be a possible explanation for the rRT-PCR positive, cELISA negative chick. The blood samples from the chicks were taken with a heparinised needle. To test if the negative cELISA results were a result of dilution of the serum, 15 of the 18 samples was retested, using a serum dilution of 1:5 (instead of 1:10), but the samples were still negative (data not shown). In contrast to our findings, ring-billed gull chicks have been found to be infected by H13 AIV within the first three weeks of life, with three-week-old chicks being 3.24 times more likely to be shedding AIV than five-week-old chicks. In addition, five-week-old chicks were 6.48 times more likely to have produced antibodies in response to AIV infection than three-week-old chicks [6].

Due to limited volumes of the sera, the samples were only tested for the H4, H5, H13 and H16 subtypes in the HI assay. The main aim was to screen the kittiwakes for antibodies to the gull-associated H13 and H16 subtypes. The specific combination of antigens (Table 1) was chosen to enable exclusion of false positives caused by neuraminidase (NA) specific antibodies in the HI assay and was the main reason why H4 and H5 were utilized in the test. In addition, the H4 was chosen because of the detection of a bird with this subtype in 2008, while H5 was selected because of the Eurasian HPAI H5N1 epidemic. The two kittiwakes that tested positive in the cELISA and negative in the HI assay could have been exposed to other subtypes than those included in the test, or the antibody level could be below the set threshold of the test. The cELISA detects antibodies to NP and has been found to be more sensitive than the HI assay that detects antibodies to HA [45].

We found that all the HI positive sera from the adult kittiwakes contained antibodies to H16, which suggests that H16 is the most common subtype of AIV in this species. This finding is in accordance with the only previous reported AIV isolate from kittiwakes [24]. In addition to antibodies to H16, a proportion of the kittiwakes also had antibodies to the H13 subtype, but the titres were lower than for H16. This indicates that H16 AIV infections are more common than H13 AIV infections in kittiwakes. The results also indirectly indicate coinfections with H16 and H13 AIVs. In Canada, 92% and 80% of the adult ring-billed gulls had antibodies to H13 AIV in 2000 and 2004, respectively, and coinfections with H16 were demonstrated [6]. The distribution of H13 and H16 AIVs in various gull species could be influenced by differences in ecological, behavioural and susceptibility factors.

## Conclusions

In conclusion, antibodies to H13 and H16 AIVs were detected in adult kittiwakes, with antibodies to H16 being most common. The serological findings suggest that black-legged kittiwake can be a reservoir for AIV and that H16 might be the main AIV subtype in this species, i.e. the world's most numerous gull species. AIV was found in low amounts in black-legged kittiwakes in the breeding season in 2008 and 2009. This is, to our knowledge, the first report of AIV in black-legged kittiwakes in Europe. Further studies are needed to understand the ecology of AIV in the black-legged kittiwake and in gulls in general.

## Competing interests

The authors declare that they have no competing interests.

## Authors' contributions

ER and RT were responsible for conception and design of the study. RT organized and conducted the field work, assisted by RTB and KB. RT performed the rRT-PCR analysis, virus isolation, hemagglutination assays and cELISAs under the guidance of AG. The HI assay was designed by CMJ, RT, AG and ER and was performed by IH. The article was drafted by RT. All authors read, revised and approved the final manuscript.
